# Editorial: Enhanced recovery after vascular surgery: state of the art and future perspectives

**DOI:** 10.3389/fsurg.2026.1852459

**Published:** 2026-05-19

**Authors:** Alberto M. Settembrini, Elena Giacomelli

**Affiliations:** 1 Saint Camillus International University of Health and Medical Sciences, Rome, Italy; 2 IRCCS MultiMedica, Sesto San Giovanni, Italy; 3 Azienda Ospedaliero Universitaria Careggi, Florence, Italy; 4 Universita Degli Studi di Firenze, Florence, Italy

**Keywords:** amputation, aorta, enhanced recovery, fast track, lower limb, thromboembolism

Enhanced Recovery After Surgery (ERAS) has changed perioperative care in surgical specialties, moving the focus from an isolated technical approach to a comprehensive, patient-centered recovery pathway. While ERAS protocols are well established in colorectal, orthopedic, and oncologic surgery, their application in vascular surgery remains heterogeneous and underdeveloped. Recent literature, including studies about abdominal aortic surgery, peripheral arterial disease, and major lower extremity amputation, underscores both the promise and importance of applying structured ERAS pathways in vascular patients to improve perioperative risk prediction, reduce vascular access complications, and increase perioperative rehabilitation. These contributions emphasize that vascular surgery is uniquely positioned to benefit from ERAS principles, given the high comorbidity burden, frailty, and prolonged recovery trajectories typical of this population (1).

Patients undergoing vascular procedures represent one of the highest-risk surgical cohorts, especially in the case of aortic surgery, peripheral arterial surgery, and lower limb amputations, because mortality, complications, and functional decline remain substantial. Major lower extremity amputation, in particular, is associated with significant morbidity, limited functional independence, and poor long-term survival. In this setting, ERAS-based multidisciplinary protocols have been proposed to address these challenges by standardizing perioperative care, optimizing preoperative assessment, implementing multimodal analgesia, and promoting early mobilization with coordinated discharge planning.

## The rationale for ERAS in vascular surgery

The vascular patient differs essentially from other surgical populations. Advanced age, diabetes, renal dysfunction, malnutrition, and frailty are common, and these factors increase postoperative complications while delaying rehabilitation. ERAS protocols aim to modify these modifiable perioperative factors. Core components, such as reduced fasting, multimodal analgesia, early mobilization, and structured discharge planning, are designed to reduce surgical stress and accelerate recovery.

The multidisciplinary nature of ERAS is particularly relevant in vascular surgery, given the complexity of these patients. Effective pathways require coordination among surgeons, anesthesiologists, nurses, physiotherapists, prosthetists, and nutritionists. Functional recovery depends not only on wound healing but also on rehabilitation, mobility restoration, and psychosocial support (2).

Intraoperative and postoperative ERAS strategies therefore focus on multimodal analgesia, including regional anesthesia and avoiding excessive opioid use, but with an improvement of the pain control to allow early mobilization, to have coordinated rehabilitation to try to reduce complications.

Among vascular procedures, major lower extremity amputation represents a paradigmatic scenario. Outcomes after amputation are determined not only by surgical technique but also by patient selection, analgesia, mobilization, and rehabilitation. Functional status and ambulation potential should be assessed early, since these factors influence postoperative rehabilitation and prosthetic use. Engagement with prosthetists prior to surgery has been associated with faster ambulation and improved outcomes, while early physiotherapy and structured discharge planning further support functional recovery and independence (Campat and O'Banion).

Early functional recovery influences long-term outcomes not only after amputation but also in uremic patients requiring dialysis access surgery. Structured rehabilitation programs, such as progressive upper-limb exercise following arteriovenous fistula creation, have demonstrated improved vascular function and better quality of life, reinforcing the importance of integrating rehabilitation into ERAS pathways (Hu et al.).

## Risk stratification and predictive modeling in ERAS pathways

Risk stratification is a central component of ERAS pathways. Predictive modeling allows tailored perioperative management and focused prevention strategies. In endovascular procedures, complications related to femoral artery access remain a major concern, affecting quality of life and healthcare costs. The identification of independent predictors—such as vascular calcification, diabetes, platelet count, and puncture site position—demonstrates the feasibility of preoperative risk prediction models (Feng et al.). By identifying high-risk patients preoperatively, clinicians can modify perioperative management, optimize access planning, adjust anticoagulation strategies, and intensify postoperative monitoring.

Similarly, ERAS implementation has been associated with reduced venous thromboembolism risk, improved laboratory markers, and lower complication rates relative to conventional care in oncologic surgery (Xu et al.). Although derived from gynecologic oncology, these findings are highly relevant to vascular surgery, where immobility, inflammation, and comorbidities increase thrombotic risk. Early mobilization, optimized anticoagulation, and structured postoperative protocols are therefore directly transferable to vascular ERAS pathways.

## The role of nursing and multidisciplinary integration

ERAS protocol can not be developed without strong support from the nurses: they are almost the central ring of the protocol. Refined nursing protocols based on ERAS principles have demonstrated improvements in clinical outcomes, including early ambulation, wound monitoring, pain management, and patient education regarding limb care. Structured perioperative education, early mobilization, and psychological support are key elements of ERAS-based nursing care. These interventions reduce complications, improve compliance, and facilitate discharge planning. The integration of nursing protocols into ERAS pathways ensures continuity of care and supports multidisciplinary collaboration (Qi et al.).

The defining feature of ERAS in vascular surgery is multidisciplinary integration across the entire perioperative continuum. Preoperative assessment identifies medical and psychosocial risks. Intraoperative management minimizes surgical stress. Postoperative care emphasizes analgesia, mobilization, and rehabilitation, while discharge planning ensures continuity of care. This integrated approach is particularly important in vascular patients, who often require long-term follow-up, prosthetic training, wound care, and risk factor modification. ERAS pathways provide a structured framework for coordinating these components and standardizing care.

## Future directions and conclusion

Future research needs to focus on procedure-specific ERAS pathways: standardized outcome measures, such as length of stay, complications, functional recovery, and quality of life, are essential for evaluating effectiveness. Additionally, digital health tools, prehabilitation programs, and frailty-based pathways may further refine ERAS implementation. The combination of predictive modeling and individualized care plans represents the next step in advancing ERAS in vascular surgery. Evidence from recent studies highlights that ERAS is not simply a perioperative protocol but a comprehensive framework for optimizing outcomes in high-risk vascular patients. The future of vascular surgery lies in the systematic adoption of ERAS pathways tailored to procedure type and patient risk profile.
